# A Case Report of a Subdural Hematoma following Spinal Epidural prior to a Total Knee Arthroplasty

**DOI:** 10.1155/2022/7548593

**Published:** 2022-06-02

**Authors:** Brian J. Carlson, David G. Deckey, Henry D. Clarke, Joshua S. Bingham

**Affiliations:** ^1^Elson S. Floyd College of Medicine, Washington State University, USA; ^2^Department of Orthopedics, Mayo Clinic Arizona, USA

## Abstract

**Introduction:**

This case report adds to current literature on management of a subdural hematoma following total knee arthroplasty and is particularly important as joint replacement moves into outpatient surgery centers where the orthopedic surgery team becomes the sole patient contact point. *Case Presentation*. A 66-year-old male presented to the emergency department five days after elective robotic-assisted left total knee arthroplasty performed with spinal epidural with the symptoms of a persistent nonpostural headache. CT of the head revealed a small bifrontal acute subdural hematoma. He was admitted for overnight monitoring as a precaution. No vascular abnormalities or underlying pathology was found on further advanced imaging. He was discharged the following morning after follow-up CT showed no focal changes. Magnetic resonance imaging (MRI) one month later confirmed resolution of the subdural hematoma.

**Conclusion:**

Orthopedic surgeons should be aware of the signs and symptoms, as well as the risk factors for subdural hematomas following lumbar puncture, as it is a rare, but potentially life-threatening complication of spinal epidural.

## 1. Introduction

Spinal anesthesia is effective and frequently utilized for lower extremity orthopedic surgery procedures and accounts for more than 50% of regional anesthesia in the spinal region [1]. Benign complications of spinal anesthesia can occur frequently, but only 0.5% of complications are critical [2, 3]. Among these critical complications are subdural hematomas (SDH) which are rare but can be serious and life-threatening. Unfortunately, the most common symptom of a SDH, a headache, is also experienced frequently after a spinal epidural as a postdural puncture headache (PDPH) [4, 5].

Epidural anesthesia offers the benefit of avoiding general anesthesia and allows patients to stay awake during the operation. Unintentional dural puncture during spinal epidural is not uncommon with a frequency of almost 4% [6]. Headaches are the most common complication after a lumbar puncture and are thought to be caused by leakage of cerebral spinal fluid (CSF) through the dural puncture causing a shift in the intracranial structure and a caudal displacement of the brain [7, 8]. A headache caused by this shift in brain position can occur in up to 33% of patients following a dural puncture [9, 10]. In some cases, this shift can lead to traction on the arachnoid mater and dural veins, which could potentially cause dural vein rupture and subsequent subdural hematoma [11]. A subdural hematoma is the most common type of intracranial hemorrhage although is still exceedingly rare [12]. The overall risk of a hemorrhagic complication after spinal anesthesia is around 1 : 220,000 [13].

Diagnosing a SDH early is essential for prevention of more serious sequela, including death. This case report presents a patient who underwent a successful and uncomplicated robotic-assisted total knee arthroplasty (TKA) who subsequently experienced a nonpostural postdural puncture headache and was subsequently hospitalized for a SDH five days postoperatively. Follow-up imaging showed resolution one month later, without any long-term complications. This case demonstrates the importance of monitoring PDPH following spinal anesthesia in lower extremity orthopedic surgery cases and highlights signs and symptoms that may necessitate follow-up and potential advanced imaging.

## 2. Case Report

**Figure 1:**
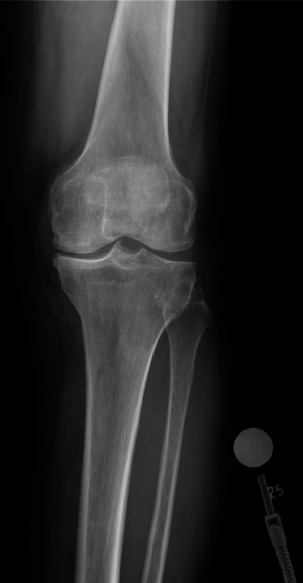
Preop anterior knee X-ray.

**Figure 2:**
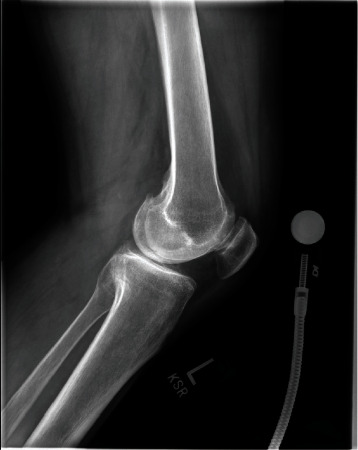
Preop lateral knee X-ray.

**Figure 3:**
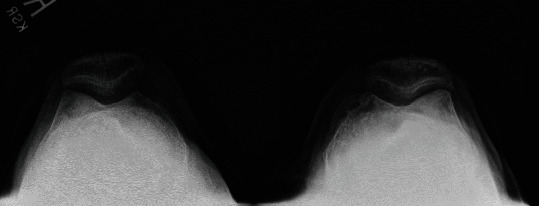
Preop merchant knee X-ray.

**Figure 4:**
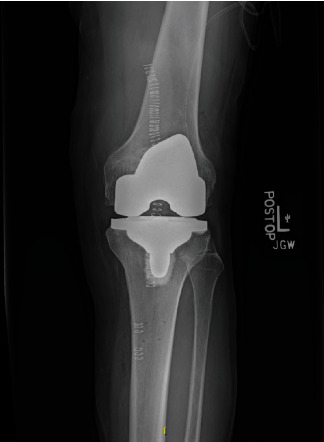
Postop AP TKA X-ray.

**Figure 5:**
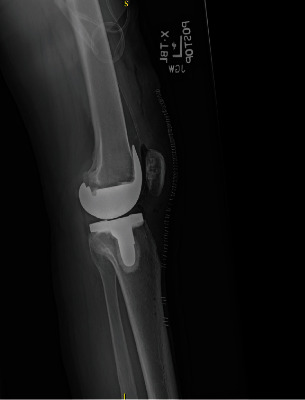
Postop lateral TKA X-ray.

**Figure 6:**
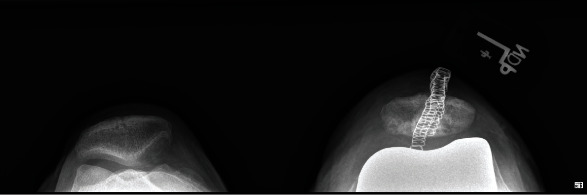
Postop merchant knee X-ray.

Informed consent was obtained from the patient, and CARE Guidelines were followed in preparation of this case report [14]. A 66-year-old otherwise healthy male was scheduled to undergo elective left robotic-assisted total knee arthroplasty for primary, end-stage osteoarthritis (Figures 1–6). He had no history of trauma, head injury, coagulation abnormality, or other pertinent past medical history. He had an American Society of Anesthesiology (ASA) score of two. The anesthesia plan was regional with a spinal epidural.

The spinal epidural was administered with the patient sitting via a midline approach at the L3-4 level by the attending anesthesiologist. It was given in a single injection on the first attempt with a 10 cm Pencan 25-gauge needle. The epidural was positive for CSF, and there was no pain with needle advancement or injection of local anesthetic. The intraoperative course was uneventful with findings of severe wear of the femoral, tibial, and patellar cartilage with predominant involvement of the medial and patellofemoral compartments. Estimated blood loss was 100 cc. The procedure lasted for 166 minutes.

Postoperatively, the patient was seen in the postanesthesia care unit and had adequate pain relief from the spinal epidural, but he did report a headache. On postoperative day 1, his pain was well-controlled; he was ambulating with physical therapy, and was subsequently discharged home. He began taking aspirin 81 mg twice daily for deep vein thrombosis prophylaxis as well as celecoxib 200 mg oral two times daily. Three days postoperatively, he called the orthopedic surgery clinic stating that his headache had worsened since surgery. He was instructed to monitor his headache and to discontinue aspirin. On postoperative day five, he was still experiencing the headache and called the COVID-19 emergency line and was advised to go to the emergency department.

**Figure 7:**
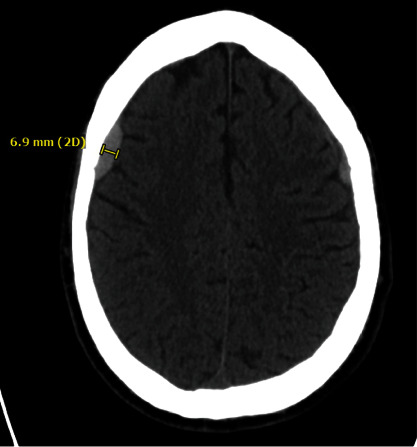
CT of left SDH at time of presentation in the emergency department.

**Figure 8:**
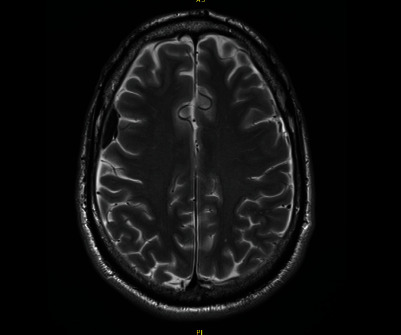
MRI showing left SDH taken at time of presentation in the emergency department.

In the emergency department, he reported that after he was discharged from this TKA, he began “hearing jet noises” while getting into his car as well as experiencing a persistent headache and tinnitus. His hearing abnormalities and constant, generalized headache were present from his time of discharge through his admittance to the emergency department. A computer tomography (CT) head scan ordered in the emergency department demonstrated a small bifrontal acute subdural hematoma. Neurosurgery was consulted for evaluation of the patient was seen by neurosurgery within 20 minutes of head CT findings (Figure 7). No vascular abnormalities or underlying pathology was found on advanced imaging. An MRI demonstrated mild dural enhancement likely related to low pressure due to the dural puncture and loss of CSF (Figure 8). He was subsequently diagnosed with a postdural puncture headache and CSF hypotension with a small subdural hematoma. He was discharged home the following day on a methylprednisolone taper with close scheduled follow-up.

**Figure 9:**
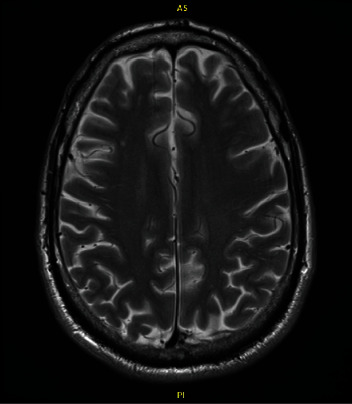
Follow-up MRI showing resolution of SDH.

At the two-week follow-up neurology visit for his admission to the emergency department, the right frontotemporal subdural hematoma had decreased in thickness and had matured to be hypodense to cortex. It had also decreased by 2 mm. The left cerebral convexity subdural hematoma had almost completely resolved. There was no evidence of new interval hemorrhage or hydrocephalus. He stated he still had a posture independent headache but that was attributed to chemical meningitis from blood breakdown. Repeat MRI one-month postdiagnosis showed that the previously noted subdural collections were no longer evident, and there were no acute focal abnormalities identified (Figure 9). He subsequently underwent a right total hip arthroplasty done under general anesthesia just over one month later with no complications. At last follow-up at 3 months, patient was doing well and was recommended to be seen again in one year for next follow-up (Table 1).

## 3. Discussion

The pathophysiology of both subdural hematomas and benign postdural puncture headaches may be similar but they have key differences [5]. In both cases, intracranial hypotension from the lumbar puncture causes a caudal shift of the brain and can cause the symptoms of a postdural puncture headache. In a postdural SDH, however, there is traction on the arachnoid mater and dural veins due to this shift. If the traction is great enough to cause a rupture of the dural veins, it can serve as the proposed mechanism for the subsequent subdural hematoma [11]. The headache that is associated with a simple PDPH has postural dependence, and the intensity of the headache will change based on body position and movement. This relationship between headache and posture, called an orthostatic headache, is present in only a handful of medical conditions and is pathognomonic for low cerebrospinal fluid due to a postdural puncture leak. Due to the similarities in presentation between a simple PDPH and more concerning subdural hematoma, clinicians should be aware of the differentiating signs and symptoms to prompt further evaluation [13]. If the headache becomes persistent, loses its relationship with body position, or changes in intensity postoperatively, a CT is to rule-out possible subdural hematoma [15].

The true incidence of subdural hematoma after dural puncture is unknown because many may resolve on their own without intervention. The true incidence may be higher than published [16]. PDPH is the most common complication of a lumbar puncture and usually resolve in a handful of days with rest and pain medication [17]. Subdural hematomas are also known to be resolved spontaneously [18]. In a cohort study of 22,130,815 postpartum women, the rate of subdural hematoma increased from 1.5 per 100,000 to 147 per 100,000 when the postdural puncture was accompanied by a headache with an overall rate of headache of 309 per 100,000 [19]. One in nine patients with a nontraumatic SDH dies during hospitalization with independent risk factors including age, blood thinner use, and coagulopathy [20].

The most common demographic to suffer from postlumbar puncture headaches are younger female patients with a lower body mass index and those who reported having headaches before the lumbar puncture [10]. One literature review found 21 cases of subdural hematoma after a spinal epidural but only two were not obstetric patients [16]. A higher body mass index (BMI > 30 kg/m^2^) has been shown to lead to a decrease incidence in PHDH with the theory that an overall increase in the epidural pressure in obese individuals decreases the pressure gradient created through loss of CSF after a dural puncture [21].

Subdural hematoma can also be a complication of spinal surgery and patients presented with symptoms anywhere between 6 hours and 50 days postoperatively and 62% of cases with cerebrospinal leaks came from the lumbar spine [22]. There are also reported cases of cervical cerebrospinal leakage causing recurrent subdural hematoma, and this possibility should be taken into consideration when a patient presents with recurrent chronic or bilateral chronic SDH [23]. There is also reported cases of acute SDH occurring in the pediatric population who had good outcomes when treated conservatively and under close observation [24].

Risk factors for a subdural hematoma include dehydration, multiple puncture holes, the use of a larger needle, use of anticoagulants, alcoholism, diabetes mellitus, and cardiovascular disease (Table 2) [13]. Subdural hematoma risk is significantly associated with antithrombotic drug use, especially vitamin K antagonist paired with an antiplatelet drug (low dose aspirin) [25]. Two studies reviewed 60 total cases of cerebral SDH following spinal anesthesia and concluded that pregnancy, dehydration, multiple dural punctures, large dural hole, use of anticoagulants, cerebral vascular abnormalities, and brain atrophy are the most common risk factors [16, 26]. Signs and symptoms to look for include a nonpostural headache with focal neurological abnormalities, an altered locus of consciousness, ptosis, plegia, and emesis. A headache lasting longer than 5 days and a headache that is unresponsive to normal treatments are also symptoms to monitor closely [27–29].

Our patient had a 25-gauge needle but studies have shown subdural hematoma with needles as small as a 27 gauge [15]. Complications can be avoided by using the smallest possible pencil-point needles, and dural leaks can be treated with autologous epidural blood patches [30].

Deaths have been recorded from subdural hematoma after lumbar puncture [15]. There is a recorded fatality from subdural hematoma after an Achilles tendon rupture repair highlighting the importance of close monitoring following surgery and early treatment when symptoms persist [15]. In order to avoid a potential poor outcome, regular follow-up of patients with headache after spinal epidural helps differentiate between a more common PDPH and a nonpostural headache, particularly in a patient with another risk factor of bleeding, such as blood thinners [15].

## 4. Conclusion

Orthopedic surgeons should be aware of the signs and symptoms as well as risk factors for a subdural hematoma following a lumbar puncture because it is a rare but potentially life-threatening complication of spinal epidural. This is particularly important as joint replacement moves into outpatient surgery centers where the orthopedic surgery team becomes the sole patient contact point. If a patient who received a spinal epidural complains of headache not related to posture or of increasing intensity in the days following surgery, a CT head scan should be ordered to rule out the possibility of SDH. Cantais et al. report a case that highlights that ignoring these signs and symptoms could potentially be fatal, even when a small needle is used. Although rare, an SDH is a life-threatening complication that can occur any time a patient undergoes a lumbar puncture and should not be confused with a PDPH.

## Figures and Tables

**Table 1:** Patient timeline.

Patient undergoes total knee arthroplasty with spinal epidural	Surgery day
Postoperatively patient reported a headache	Surgery day
Patient was discharged home	Postop day 1
Patient calls orthopaedic surgery clinic reporting consistent headache	Postop day 3
Patient calls COVID-19 line and is advised to go to the emergency department	Postop day 5
CT imaging showed bilateral SDH	Postop day 5
Patient was discharged home on methylprednisone	Postop day 6
Follow-up imaging showed a 2 mm reduction in SDH	Postop day 20
Subsequent follow-up imaging showed resolution of SDH	Postop day 35
Patient underwent right total hips arthroplasty	Postop day 65
Patient seen at three-month follow-up with no complications	

**Table 2:** 

Risk factors for subdural hematoma
Dehydration
Multiple puncture holes
Use of a larger needle
Use of anticoagulants
Alcoholism
Diabetes mellitus
Cardiovascular disease
